# Facile Synthesis of Iron and Nitrogen Co-Doped Carbon Dot Nanozyme as Highly Efficient Peroxidase Mimics for Visualized Detection of Metabolites

**DOI:** 10.3390/molecules28166064

**Published:** 2023-08-15

**Authors:** Shuai Xu, Shiyue Zhang, Yutong Li, Jiyang Liu

**Affiliations:** School of Chemistry and Chemical Engineering, Zhejiang Sci-Tech University, Hangzhou 310018, China; 202120104178@mails.zstu.edu.cn (S.X.); 202120104186@mails.zstu.edu.cn (S.Z.); 202030107261@mails.zstu.edu.cn (Y.L.)

**Keywords:** visual detection, colorimetric sensor, nanozyme, carbon dots, metabolite detection

## Abstract

Visual detection based on nanozymes has great potential for the rapid detection of metabolites in clinical analysis or home-based health management. In this work, iron and nitrogen co-doped carbon dots (Fe,N-CDs) were conveniently synthesized as a nanozyme for the visual detection of glucose (Glu) or cholesterol (Chol). Using inexpensive and readily available precursors, Fe,N-CDs with peroxidase-like activity were conveniently prepared through a simple hydrothermal method. Co-doping of Fe and N atoms enhanced the catalytic activity of the nanozyme. The nanozyme had a low Michaelis constant (*K*_m_) of 0.23 mM when hydrogen peroxide (H_2_O_2_) was used as the substrate. Free radical trapping experiments revealed that the reactive oxygen species (ROS) generated in the nanozyme-catalyzed process were superoxide anion radicals (•O^2−^), which can oxidize colorless 3,3′,5,5′-tetramethylbenzidine (TMB) to generate blue oxidation product (ox-TMB) with characteristics absorbance at 652 nm. Based on this mechanism, a colorimetric sensor was constructed to detect H_2_O_2_ ranging from 0.1 μM to 200 μM with a detection limit (DL) of 75 nM. In the presence of glucose oxidase (Gox) or Chol oxidase (Chox), Glu or Chol was oxidized, respectively, and generated H_2_O_2_. Based on this, indirect detection of Glu and Chol was realized with linear detection ranges of 5–160 μM and 2–200 μM and DLs of 2.8 μM and 0.8 μM, respectively. A paper-based visual detection platform was fabricated using Fe,N-CDs as nanozyme ink to prepare testing paper by inkjet printing. Using a smartphone to record the RGB values of the testing paper after the reaction, visual detection of Glu and Chol can be achieved with linear detection ranges of 5–160 μM (DL of 3.3 μM) and 2–200 μM (DL of 1.0 μM), respectively.

## 1. Introduction

Metabolite detection provides vital health information, enabling early disease detection and guiding treatment, while also playing an important role in individual health management [1,2,3,4,5]. For example, Glu is a necessary nutrient for metabolism in organisms [6,7]. In the body, Glu is oxidized into carbon dioxide and water, while also providing energy and being stored in the form of glycogen. Abnormal blood Glu levels are an important factor in triggering serious health problems such as cardiovascular diseases and diabetes. On the other hand, Chol is primarily synthesized by the human body [8]. Under normal circumstances, the body synthesizes Chol in the liver and obtains it from food. Chol can be converted into steroid hormones or become a component of cell membranes, while also maintaining stable Chol levels in the blood. When the liver experiences severe disease, Chol levels can decrease. However, in patients with obstructive jaundice and nephrotic syndrome, Chol levels often increase. Therefore, effective and convenient monitoring of the levels of Glu and Chol in the human body is of great significance in the early prevention and treatment of diseases.

Visual detection of metabolites provides an intuitive, accurate, and real-time method to understand changes in metabolic processes within organisms, playing a significant role in promoting disease diagnosis and monitoring, drug development and evaluation, as well as advancing personalized medicine and health management [9,10,11,12,13]. With the widespread adoption of smartphones, visual detection based on mobile phones has gained increasing attention in clinical analysis and home health management [14,15,16]. Firstly, phone-based visual detection offers convenience as it can be performed anytime without time or location limitations. Users can simply use a smartphone for detection without relying on special equipment or professional support. Secondly, phone-based visual detection allows for real-time monitoring of biological parameters and health indicators. Additionally, the detection results can be stored on the phone or in the cloud, enabling users to conveniently access and manage their health data at any time. Moreover, users can share the data with doctors or healthcare professionals for remote monitoring and diagnosis. Therefore, the development of simple and user-friendly methods for phone-based visual detection of metabolites holds great significance.

Currently, enzyme-catalyzed detection is the most commonly used method for metabolite detection [17,18,19,20]. For example, glucose oxidase (Gox) is used to catalyze the reaction between Glu and a co-enzyme (such as NAD^+^ or FAD), generating H_2_O_2_, gluconic acid, and the reduced form of the co-enzyme. Chol detection often employs cholesterol oxidase to catalyze the reaction between Chol and the corresponding co-enzyme, also producing H_2_O_2_, cholest-4-en-3-one, and the reduced form of the co-enzyme. By measuring the current, absorbance, or fluorescence signal of the produced H_2_O_2_ or reduced co-enzyme, the concentration of Glu or Chol in the sample can be determined. However, natural enzymes as traditional biocatalysts have limitations, such as easy deactivation in extreme environments, high preparation cost, and difficulty in long-term storage. Therefore, in recent years, many studies have focused on developing stable and efficient artificial enzymes as substitutes for natural enzymes [21]. Nanozymes are a class of nanomaterials with enzyme-like catalytic activity and enzymatic characteristics, possessing advantages such as tunable structure and high stability [22]. Consequently, the development of novel nanozymes with simple synthesis and high catalytic performance is highly desirable. With the development of functional nanomaterials, nanocarbon materials with different dimensions, such as three-dimensional (3D) [23,24,25] or two-dimensional (2D) graphene [26,27,28,29], and zero-dimensional (0D) carbon dots (CDs) or graphene quantum dots (GQDs) [30,31], have garnered significant attention due to their widely adjustable structures, excellent electrical or optical properties, high stability, and good biocompatibility. Among them, CDs exhibit tunable composition, remarkable fluorescence, low cost, etc. [32,33,34,35]. Recently, it has been proven that doping with a heteroatom (e.g., Fe,N) can endow CDs with high nanozyme activity. CD-based nanozymes have demonstrated potential applications in the fields of biological analysis, medical detection, environmental analysis, and so on [36,37].

In this work, a one-step hydrothermal method was established to synthesize iron and nitrogen co-doped carbon dots (Fe,N-CDs) with high peroxidase-like activity. The as-prepared Fe,N-CDs can catalyze the oxidation of colorless TMB to blue ox-TMB in the presence of H_2_O_2_, leading to colorimetric detection of H_2_O_2_. By introducing Gox or Chox, Glu or Chol can be converted into H_2_O_2_, enabling colorimetric detection of Glu and Chol. Based on the water solubility and peroxidase-like activity of Fe,N-CDs, paper-based sensors were easily fabricated through simple inkjet printing by using nanozymes as ink. The color of the paper-based sensor was recorded using a smartphone to read the RGB values, leading to visual detection of Glu or Chol.

## 2. Results and Discussion

### 2.1. Strategy for One-Pot Synthesis of Fe,N-CDs and Colorimetric Detection of Metabolism

**Figure 1:**
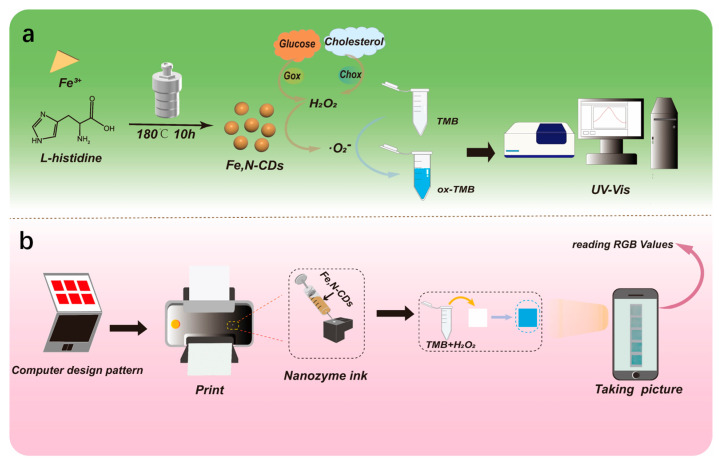
(**a**) Schematic illustration of the one-step preparation of Fe,N-CDs and colorimetric detection of Glu and Chol. (**b**) Illustration of the preparation of testing paper using nanozymes as ink through inkjet printing for visual detection.

CDs possess several advantages, including adjustable size, excellent optical properties, biocompatibility, and chemical modifiability [38,39,40]. Figure 1a illustrates the synthesis of Fe,N-CDs using a one-pot hydrothermal method for colorimetric detection. Inexpensive and easily accessible histidine and ferric chloride were utilized as raw materials to synthesize Fe,N-CDs through a bottom-up approach. The resulting yield of Fe,N-CDs from this method is 7.4%. Fe,N-CDs exhibit peroxidase-like activity and can catalyze the oxidation of colorless TMB to blue oxides of TMB (ox-TMB) by H_2_O_2_. Thus, the detection of H_2_O_2_ can be achieved by measuring the characteristic absorption of ox-TMB using UV-visible spectroscopy. As is known, H_2_O_2_ is an intermediate product of metabolism. For example, the oxidation of Glu or Chol catalyzed by Gox or Chox can generate H_2_O_2_. Therefore, colorimetric detection of H_2_O_2_ enables indirect detection of Glu or Chol.

Visual detection is a straightforward and user-friendly method that does not require specialized equipment or technical expertise. It yields immediate results, enabling fast and convenient testing, particularly for on-site or home monitoring purposes. Figure 1b illustrates the process of preparing testing paper using nanozyme ink for visually detecting H_2_O_2_. As shown, nanozymes can be directly used as ink with a commonly available inkjet printer to create the testing paper. After placing the testing paper containing nanozymes into a mixture solution of TMB substrate and different concentrations of H_2_O_2_ for reaction, a smartphone can capture images of the reacted testing paper. H_2_O_2_ detection can be achieved by extracting the RGB values using Image J software. As inkjet printing for the preparation of testing paper offers several advantages, including precise control, efficient production, easy integration of multiple functions, and cost-effectiveness, the fabricated paper-based sensor exhibits significant potential for rapid, large-scale, and customized testing paper production.

### 2.2. Characterization of Fe,N-CDs

**Figure 2:**
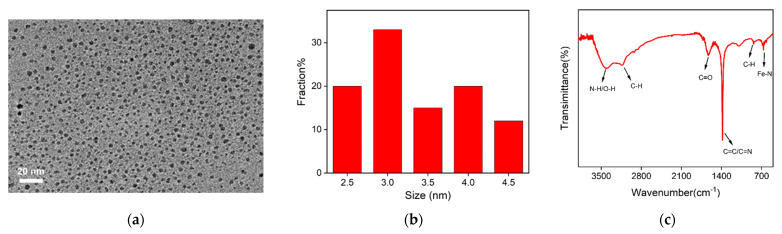
TEM image (**a**), size distribution plot (**b**), and FT-IR spectrum of Fe,N-CDs (**c**).

The size of Fe,N-CDs was characterized by TEM. From the TEM image (Figure 2a), it can be observed that Fe,N-CDs are well dispersed without aggregation, and the size is relatively uniform. The particle size distribution shows that the size of Fe,N-CDs ranges from 2.5 nm to 4.5 nm, with an average size of 3.4 nm (Figure 2b).

The functional groups of Fe,N-CDs were characterized by FT-IR. As shown in Figure 2c, the broad absorption peak at 3425 cm^−1^ and the weaker absorption peak at 3132 cm^−1^ can be attributed to the stretching vibration of N-H/O-H and the characteristic absorption peak of C-H, respectively. The strong absorption peaks at 1634 cm^−1^ and 1384 cm^−1^ are attributed to the stretching vibration of C=O and C=C/C=N, respectively. These results indicate that Fe,N-CDs contain abundant oxygen and nitrogen functional groups, which contribute to their good dispersion in aqueous solutions. The peak observed at 680 cm^−1^ corresponds to the stretching vibration of Fe-N, indicating the successful doping of Fe atoms into the carbon framework of the CDs.

**Figure 3:**
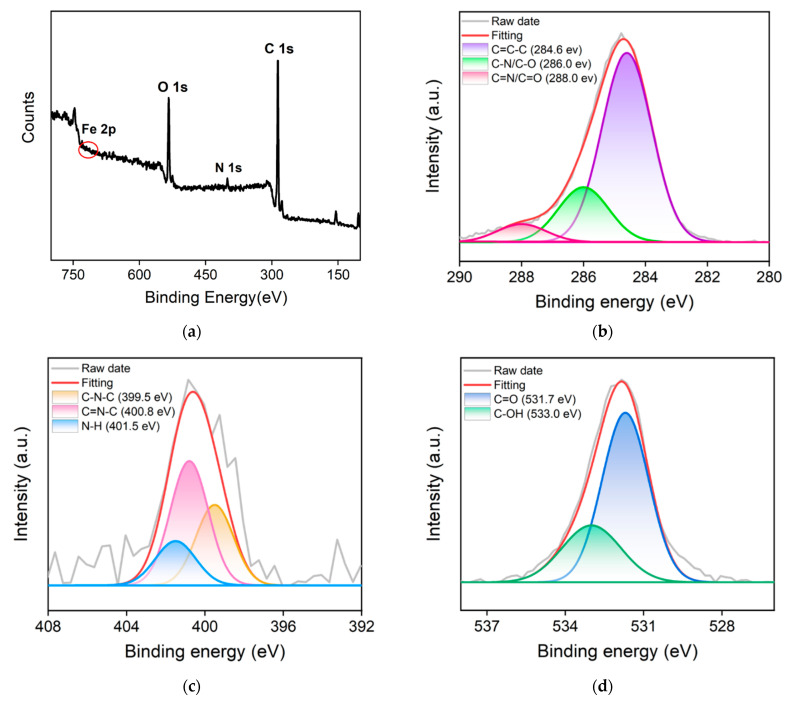
(**a**) XPS survey spectrum of Fe,N-CDs. High-resolution C1s (**b**), N1s (**c**), and O1s (**d**) spectra of Fe,N-CDs.

The element composition and chemical groups of Fe,N-CDs were further characterized by XPS. Figure 3a shows the XPS survey spectrum of Fe,N-CDs. As seen, Fe,N-CDs mainly consist of three elements, C, N, and O, with corresponding percentages of 78.7%, 3.2%, and 17.8%, respectively. In addition, a very low signal of Fe atoms can be observed, accounting for 0.3% of the total composition. Furthermore, the Fe content in Fe,N-CDs was determined by ICP-OES, and 5.6% of Fe is revealed. The difference in the Fe content measured by the two methods is attributed to the fact that XPS can only detect the surface Fe atoms of Fe,N-CDs. Figure 3b presents the high-resolution C 1s spectrum. The signals with binding energies at 284.6 eV, 286.0 eV, and 288.0 eV correspond to graphitic C (C-C=C, sp^2^ carbon), C-N/C-O bond, and C=N/C=O bond, respectively. The high-resolution N 1s spectrum in Figure 3c shows peaks at binding energies of 399.5 eV, 400.8 eV, and 401.5 eV, corresponding to C-N-C, C=N-C, and N-H structures, respectively. The high-resolution O 1s spectrum in Figure 3d exhibits peaks at binding energies of 531.7 eV and 533.0 eV, corresponding to C=O and C-OH, respectively. These results further confirm the presence of functional groups such as imidazole, carbonyl, and hydroxyl groups on the surface of Fe,N-CDs.

### 2.3. Peroxidase-like Activity of Fe,N-CDs

**Figure 4:**
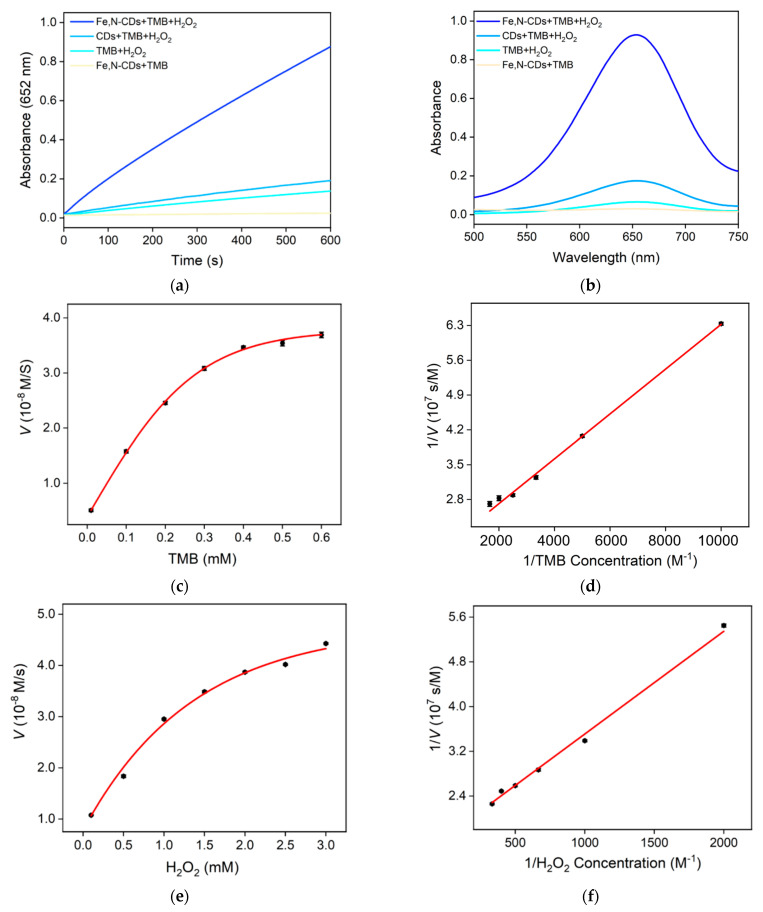
(**a**) Time-dependent absorbance changes at 652 nm for different mixed solutions. (**b**) UV–visible spectra of different mixed solutions after a 10-minute reaction. (**c**) Relationship between TMB concentration and reaction rate at a fixed H_2_O_2_ concentration. (**d**) Double reciprocal plot of Fe,N-CDs activity obtained by changing the TMB concentration using the Michaelis–Menten model. (**e**) Relationship between H_2_O_2_ concentration and reaction rate at a fixed TMB concentration. (**f**) Double reciprocal plot of Fe,N-CDs activity obtained by changing the H_2_O_2_ concentration.

The peroxidase-like activity of Fe,N-CDs can be examined using TMB as a substrate, which is a commonly used substrate to evaluate the enzymatic activity of peroxidase-like nanozymes. Commonly, nanozymes can catalyze the oxidation of TMB by H_2_O_2_ to form blue ox-TMB. The higher the absorbance of ox-TMB, the higher the nanozyme activity. Figure 4a shows the time-dependent changes in absorbance at 652 nm (A_652 nm_) for different reaction solutions. For comparison, CDs with only N doping (N-CDs), synthesized with only L-histidine as a precursor, were applied as the control. As seen, the addition of N-CDs resulted in slight increases in absorbance. In the presence of Fe,N-CDs, a significantly higher increase in absorbance is observed, indicating higher nanozyme activity. Figure 4b shows the absorption spectra of different reaction solutions after a 10-min reaction. As seen, the absorbance of the Fe,N-CDs + TMB + H_2_O_2_ solution is significantly higher than that of the other groups. These results indicate that Fe,N-CDs exhibit the highest nanozyme activity, which can be attributed to the co-doping of Fe and N atoms [40,41,42,43]. Doping nitrogen and iron atoms into CDs might alter their band structure and energy level distribution. On the one hand, N atoms establish robust covalent bonds with carbon atoms, improving electrical conductivity and enhancing chemical stability by increasing electron density. On the other hand, Fe elements play an important role in the catalytical property of natural peroxidase. The introduction of Fe atoms might increase active sites on the surface of CDs and facilitate electron transfer during catalysis, thereby enhancing substrate adsorption capacity and improving catalytic reaction rate and efficiency.

The enzymatic performance of Fe,N-CDs as a peroxidase mimic was evaluated by determining the steady-state kinetic constants. The Michaelis–Menten curves were obtained by using TMB and H_2_O_2_ as substrates, and the Michaelis constant (*K*_m_) and maximum initial reaction rate (*V*_max_) values were calculated using the Lineweaver–Burk plot. When TMB is used as the substrate, the *K*_m_ value is determined to be 0.23 mM, and the *V*_max_ is 5.510 × 10^−8^ M/s (Figure 4c,d). When H_2_O_2_ is used as the substrate, the *K*_m_ value is 1.10 mM, and the *V*_max_ is 5.598 × 10^−8^ M/s (Figure 4e,f).

### 2.4. Catalytic Mechanism of Fe,N-CD Nanozyme

**Figure 5:**
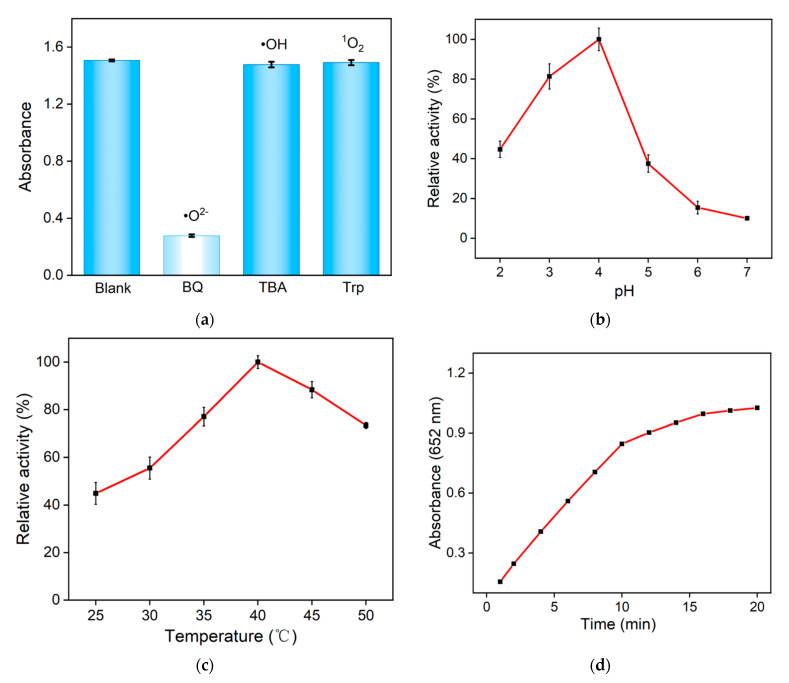

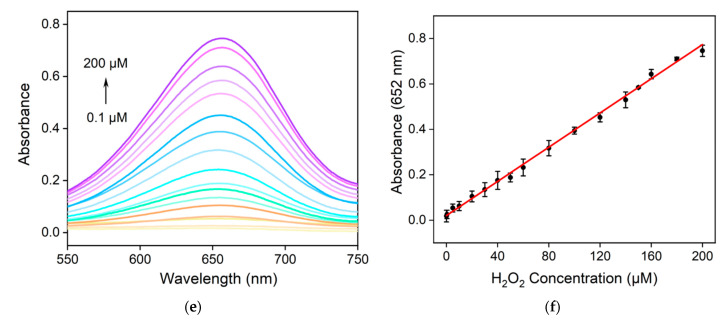
(**a**) The absorbance values at 652 nm of the products obtained from the catalytic oxidation of TMB by Fe,N-CDs in the presence of different ROS scavengers. The effects of pH (**b**), reaction temperature (**c**), and reaction time (**d**) on the catalytic reaction. (**e**) Absorption spectra of the mixed solution after adding different concentrations of H_2_O_2_ to TMB and Fe,N-CDs. (**f**) Linear relationship plot between A_652_ and H_2_O_2_ concentration.

The catalytic mechanism of Fe,N-CDs was investigated by adding different scavengers to capture ROS generated during the Fe,N-CDs catalytic process. The scavengers used for capturing different free radicals were benzoquinone (BQ) for •O^2−^, tryptophan (Trp) for singlet oxygen (^1^O_2_), and tert-butanol (TBA) for hydroxyl radical (•OH). When free radicals are captured by scavengers, the production of ox-TMB might significantly decrease, leading to a decrease in absorbance. As shown in Figure 5a, the addition of BQ results in a sharp decrease in absorbance, indicating that Fe,N-CDs mainly generate •O^2−^ as the ROS during nanozyme-catalyzed H_2_O_2_ decomposition.

### 2.5. Colorimetric Detection of H_2_O_2_

Fe,N-CDs exhibit nanozyme activity and can catalyze the decomposition of H_2_O_2_ to generate ROS, which further oxidizes colorless TMB to produce blue-colored ox-TMB. Thus, the detection of H_2_O_2_ can be achieved by the quantification of ox-TMB. To obtain the optimal conditions for detecting H_2_O_2_, the effects of reaction pH, temperature, and reaction time on the catalytic reaction were investigated. As shown in Figure 5b, Fe,N-CDs exhibit the highest peroxidase-like activity at pH 4.0. At a reaction temperature of 40 °C, Fe,N-CDs show the highest catalytic activity, which is attributed to the fact that both the Fe,N-CD nanozyme and natural enzymes have the highest activity near physiological temperatures (Figure 5c). Therefore, the subsequent experiments were conducted at physiological temperatures for detection. When TMB, H_2_O_2_, and Fe,N-CDs reacted for 10 min, the reaction reached equilibrium (Figure 5d). The optimized conditions mentioned above were used for subsequent experiments.

Figure 5e shows the absorption spectra of the solution after adding different concentrations of H_2_O_2_ to TMB and Fe,N-CDs, followed by a 10-minute reaction. It can be observed that the absorbance values of the solution increase with increasing H_2_O_2_ concentration. As shown in Figure 5f, the absorbance values at 652 nm (A_652_) exhibit a good linear relationship with H_2_O_2_ concentration (C) in the range of 0.1–200 μM (A_652_ = 0.00377C + 0.0200, R^2^ = 0.998). The detection limit (DL) calculated based on a signal-to-noise ratio of three (S/N = 3) is 75 nM.

### 2.6. Colorimetric Detection of Glu and Chol

**Figure 6:**
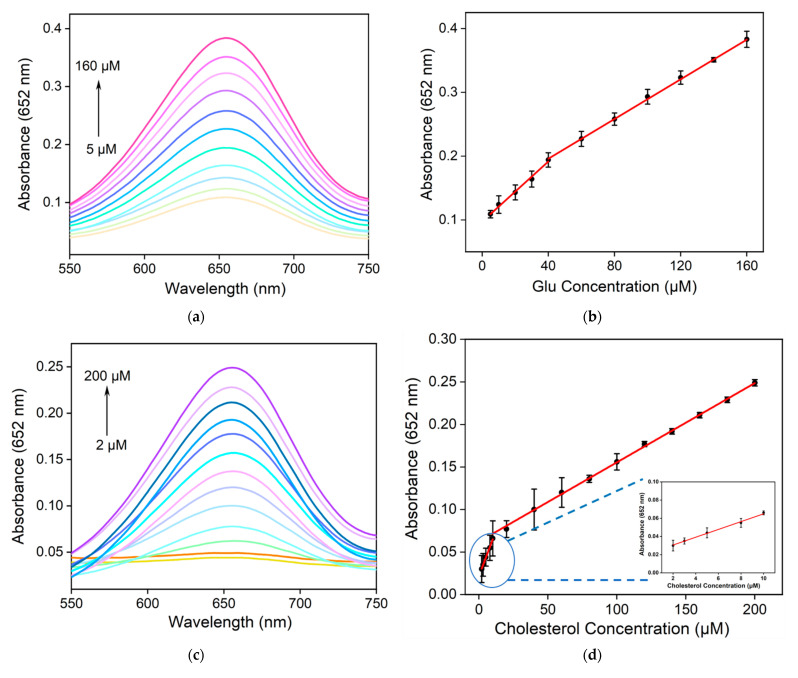

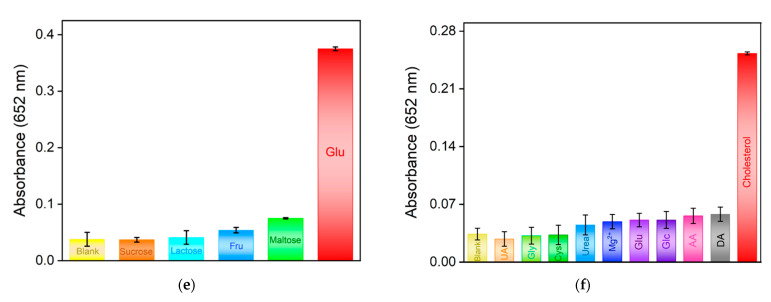
(**a**) Absorption spectra of the mixed solution after adding different concentrations of Glu to TMB, Fe,N-CDs, and Gox. (**b**) Linear relationship plot between A_652_ and Glu concentration. (**c**) Absorption spectra of the mixed solution after adding different concentrations of Chol to TMB, Fe,N-CDs, and Chox. (**d**) Linear relationship plot between A_652_ and Chol concentration. (**e**) A_652_ of the solution after adding Glu (200 μM) or other substances (1 mM). (**f**) A_652_ of the solution after adding Chol (200 μM) or other substances (1 mM).

The detection of metabolites such as Glu and Chol is of significant importance in the diagnosis and treatment of chronic diseases such as diabetes and dyslipidemia. In the presence of Gox and Chox, Chol and Glu can be converted to H_2_O_2_. Therefore, indirect detection of Glu or Chol can be achieved through quantitatively measuring H_2_O_2_. Figure 6a shows the absorption curves of the mixed solution containing Fe,N-CDs, TMB, and Gox after adding different concentrations of Glu. As seen, the absorbance of the solution increases with increasing Glu concentration. As shown in Figure 6b, there is a linear relationship between A_652_ and Glu concentration within the ranges of 5–40 μM (A_652_ = 0.0023C_Glu_ + 0.097, R^2^ = 0.996) and 40–160 μM (A_652_ = 0.0015C_Glu_ + 0.13, R^2^ = 0.999). The DL is 2.8 μM (S/N = 3). Similar to the mechanism described above, Chol is also converted into H_2_O_2_ in the presence of Chox. As shown in Figure 6c, the absorbance of the mixed solution increases with increasing Chol concentration. In Figure 6d, A_652_ exhibits a linear relationship with Chol concentration within the ranges of 2–10 μM (A_652_ = 0.004C_Chol_ + 0.023, R^2^ = 0.996) and 10–200 μM (A_652_ = 0.001C_Chol_ + 0.059, R^2^ = 0.998). The DL is 0.8 μM (S/N = 3). As seen, the sensitivity for the Glu or Chol detection decreases at a high concentration range. Such a phenomenon is commonly observed in many sensors, attributable to decreased active binding sites and mass transfer.

Selectivity is an important parameter for evaluating a sensor. To investigate the selectivity of the constructed colorimetric sensor for Glu detection, several common sugars such as sucrose, lactose, fructose (Fru), and maltose were selected as possible interfering substances. As shown in Figure 6e, even when five times the concentration of other sugars was added, there was no significant increase in absorbance, indicating that the sensor has good selectivity for Glu. The selectivity of the colorimetric sensor for Chol detection was also evaluated by adding common interferents in serum, such as uric acid (UA), urea, cysteine (Cys), glycine (Gly), MgCl_2_, glutamic acid (Glc), Glu, ascorbic acid (AA), and dopamine (DA). As shown in Figure 6f, a significant change in the signal is observed even with a five times concentration of interferents, demonstrating the excellent selectivity of the sensor.

The content of Glu and Chol in serum was detected using the standard addition method. As shown in Table 1, satisfactory recovery rates were obtained for Glu or Chol detection (96.0–98.8% for Glu detection, 97.5–104% for Chol detection). The relative standard deviation (RSD) of the measurements is no higher than 2.2%, indicating good accuracy of the detection.

### 2.7. Visual Detection of Glu and Chol Using Nanozyme Paper and Smartphone

**Figure 7:**
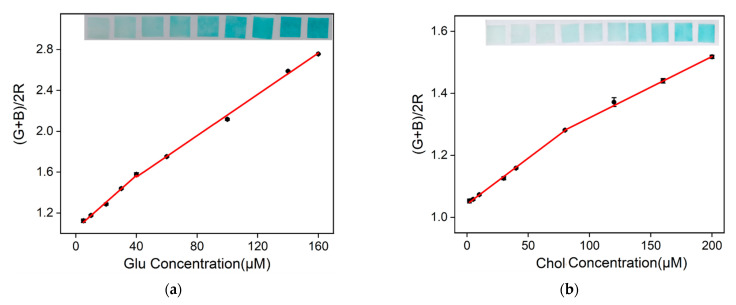
The linear relationship between (G + B)/2R and (**a**) Glu and (**b**) Chol concentrations is shown in the graph. The inset images are photographs of the test strips, with increasing analyte concentrations from left to right.

Smartphone-based visual detection offers advantages such as convenience, simplicity, real-time results, remote monitoring, and multi-functionality. Nanozyme testing paper can be conveniently prepared using inkjet printing, serving as a paper-based sensor and enabling the visual detection of Glu or Chol. As shown in the insets of Figure 7a, the blue color of the testing paper intensifies as the concentration of the analyte increases. The sum of green and blue chromaticity values divided by twice the red chromaticity value [(G + B)/2R, designated as y] exhibits good linearity with Glu concentration within the ranges of 5–40 μM (y = 0.0013 *C*_Glu_ + 1.04, *R*^2^ = 0.996) and 40–160 μM (y = 0.0010 *C*_Glu_ + 0.98, *R*^2^ = 0.998). The detection limit is 3.3 μM (S/N = 3). Similarly, as shown in Figure 7b, [(G + B)/2R] shows good linearity with Chol concentration within the ranges of 2–80 μM (y = 0.0029 *C*_Chol_ + 1.04, *R*^2^ = 0.999) and 80–200 μM (y = 0.0020 *C*_Chol_ + 1.12, *R*^2^ = 0.999). The detection limit is 1.0 μM.

## 3. Materials and Methods

### 3.1. Chemicals and Materials

L-cysteine, tryptophan, fructose, quinone, Glu, ascorbic acid, magnesium sulfate, tert-butanol, 3,3′,5,5′-tetramethylbenzidine (TMB), 5,5-dimethyl-1-pyrroline N-oxide (DMPO), iron(III) chloride, lactose, sucrose, maltose, urea, and glycine were purchased from Aladdin Biochemical Technology Co., Ltd. (Shanghai, China). Uric acid, dopamine, Chol, and Chol oxidase (Chox) were obtained from Shanghai Macklin Biochemical Co., Ltd. (Shanghai, China). Glu oxidase (Gox) was purchased from Sigma-Aldrich (Shanghai, China) Trading Co., Ltd. (Shanghai, China). Hydrogen peroxide was purchased from Tianjin Yongda Chemical Reagent Co., Ltd. (Shanghai, China), and methanol was obtained from Hangzhou Gaojing Fine Chemical Co., Ltd. (Hangzhou, China). All reagents used in the experiments are of analytical purity and have not undergone additional purification. Ultrapure water with a resistivity of 18 MΩ·cm was employed.

### 3.2. Characterizations and Instrumentations

The size and morphology of CDs were examined using a transmission electron microscope (TEM, JEM-2100, JEOL Ltd., Tokyo, Japan) with a testing voltage of 200 kV, using ultrathin carbon film as the substrate. X-ray photoelectron spectroscopy (XPS, PHI5300, Physical Electronics, Boston, MA, USA) and Fourier-transform infrared spectroscopy (FT-IR, Vertex 70, Bruker Corporation, Karlsruhe, Germany) were employed to investigate the functional groups of CDs. XPS measurements were performed under the conditions of 250 W, 14 kV, and with Mg Kα radiation. The UV–Vis absorption spectrum of Fe,N-CDs was recorded using a UV-2450 UV-Visible Spectrophotometer (UV-Vis, Shimadzu Corporation, Tokyo, Japan). The particle size distribution of CDs was determined using a particle size analyzer (SZ-100V2, Horiba Corporation, Tokyo, Japan). The iron content in CDs was determined using an inductively coupled plasma optical emission spectrometer (ICP-OES, Agilent 730, Agilent Technologies, Palo Alto, CA, USA).

### 3.3. One-Step Synthesis of Fe,N-CD Nanozyme

Fe,N-CDs were synthesized using a one-pot hydrothermal method. Briefly, 0.1 g of L-histidine and 0.1 g of anhydrous ferric chloride were added to 20 mL of ultrapure water. The resulting mixture was stirred at room temperature for 30 min before being transferred to a reaction vessel lined with polytetrafluoroethylene (PTFE). The reaction was carried out at 180 °C for 10 h. The resulting solution was first filtered through a 0.22 μm membrane to remove large particles, and then dialyzed for 48 h using a dialysis bag with a cut-off molecular weight of 500 Da. The liquid inside the dialysis bag was freeze-dried to obtain solid Fe,N-CDs.

### 3.4. Enzyme Activity Testing of Fe,N-CD Pseudo-Peroxidase

The catalytic behavior of the Fe,N-CD nanozyme was studied using 3,3′,5,5′-tetramethylbenzidine (TMB) and H_2_O_2_ as substrates. The experiment was carried out in NaAc-HAc buffer solution (0.1 M, pH 4.0), with the addition of Fe,N-CDs (60 μg/mL) for the test group and non-doped CDs (60 μg/mL) for the control group. After incubation in a constant temperature water bath at 40 °C for 10 min, the UV absorption spectra of the reaction system were recorded. Subsequently, the kinetics parameters of Fe,N-CDs were studied using the Michaelis–Menten model. When H_2_O_2_ was applied as substrate, the H_2_O_2_ concentration was varied from 0.1 mM to 3 mM, and the TMB concentration was fixed at 0.5 mM under optimal pH conditions. In the case of the TMB substrate, the concentration of H_2_O_2_ was fixed at 0.2 mM, while the TMB concentration was varied from 0.01 mM to 0.6 mM. The changes in absorbance at 652 nm over time were recorded for 10 min. Finally, the *K*_m_ and Vmax of Fe,N-CDs were calculated using the reciprocal of the double reciprocal plot according to the Lineweaver–Burk equation.

### 3.5. Detection of Free Radicals Generated during the Catalytic Process of Fe,N-CDs

The scavengers used for capturing different free radicals were benzoquinone for •O^2−^, tryptophan for singlet oxygen (^1^O_2_), and tert-butanol for hydroxyl radical (•OH). Using TMB as the substrate, the absorbance values were measured after 10 min of reaction in the TMB + Fe,N-CDs + H_2_O_2_ system with the addition of the three free radical scavengers mentioned above. The final concentrations of TMB, Fe,N-CDs, and H_2_O_2_ were 0.5 mM, 60 μg/mL, and 6.6 mM, respectively. The concentrations of benzoquinone, tryptophan, and tert-butanol were 100 μM and 100 μg/mL, respectively. Further validation of the experimental results was conducted through ESR testing, using DMPO + H_2_O_2_ or DMPO + H_2_O_2_ + Fe,N-CD solutions. The final concentrations of DMPO, H_2_O_2_, and Fe,N-CDs were 20 mM, 20 mM, and 100 μg/mL, respectively.

### 3.6. Colorimetric Detection of H_2_O_2_

A single-factor optimization method was employed to optimize the temperature, pH value, reaction time, and Fe,N-CD concentration in the colorimetric detection of H_2_O_2_. Under optimal conditions, the detection of H_2_O_2_ was performed. Briefly, different concentrations of H_2_O_2_ were added to a solution containing NaAc-HAc buffer (0.1 M, pH 4.0), TMB (0.5 mM), and Fe,N-CDs (60 μg/mL). After incubation at 37 °C for 10 min, the UV absorption spectra of the solution were recorded.

### 3.7. Colorimetric Detection of Glu

Colorimetric detection of Glu was performed in the presence of Fe,N-CDs and glucose oxidase. In simple terms, different concentrations of Glu were added to a mixed solution containing NaAc-HAc buffer (0.1 M, pH 4), TMB (0.5 mM), Fe,N-CDs (60 μg/mL), and Gox (0.1 mg/mL). After incubating at 37 °C for 10 min, the UV absorption spectra of the solution were recorded. To investigate the selectivity of the constructed colorimetric sensor for detecting Glu, several common sugars such as sucrose, maltose, fructose, and lactose were chosen as interfering substances. The changes in absorbance of the above detection solution were measured when the concentration of each sugar substance (1 mM) was 5 times the concentration of Glu. To further explore the potential application of the constructed colorimetric sensor in real sample analysis, Glu content in bovine serum (diluted 50 times) was determined using the standard addition method.

### 3.8. Colorimetric Detection of Chol

Colorimetric detection of Chol was achieved using Fe,N-CDs and cholesterol oxidase. Briefly, different concentrations of Chol were added to a mixed solution containing NaAc-HAc buffer (0.1 M, pH 4), TMB (0.5 mM), Fe,N-CDs (60 μg/mL), and Chox (0.05 mg/mL). After incubating at 37 °C for 10 min, the UV absorption spectra of the solution were recorded. To explore the selectivity of the sensor for Chol detection, uric acid, urea, cysteine, glycine, MgSO_4_, glutamic acid, Glu, ascorbic acid, and dopamine were used as the possible interfering substances, and the changes in absorbance of the detection solution were measured when each substance had a concentration of 1 mM. The determination of Chol in bovine serum (diluted 50 times) was also investigated using the standard addition method.

### 3.9. Visual Detection of H_2_O_2_, Glu, or Chol Based on Nanozyme Testing Paper

Nanozyme testing paper was prepared using an inkjet printer (HP Deskjet 1112, HP Inc., Palo Alto, CA, USA). Fe,N-CDs were used as ink (0.5 mg/mL) and filter paper as the printing medium. The printed filter paper was cut into 2 cm × 2 cm pieces for further investigation. For the detection of H_2_O_2_, different concentrations of H_2_O_2_ were mixed with NaAc-HAc buffer (0.1 M, pH 4.0) and TMB (0.5 mM). Then, 200 μL of the mixture was dropped onto the paper-based sensor, followed by incubation at 37 °C for 30 min. The color of the testing paper was recorded by taking a photo using a smartphone. For detecting Glu, glucose oxidase was used. Briefly, different concentrations of Glu were mixed with NaAc-HAc buffer (0.1 M, pH 4.0), TMB (0.5 mM), and Gox (0.1 mg/mL) Similarly, 200 μL of the mixture was dropped onto the paper-based sensor, followed by incubation at 37 °C for 30 min. The color of the testing paper was then recorded using a smartphone. Subsequently, the red–green–blue (RGB) values of the testing paper were analyzed using Image J software. The visual detection of Chol was performed using the same procurement when Gox was replaced with Chox (0.05 mg/mL).

## 4. Conclusions

In this work, Fe,N-CDs were synthesized through a one-step hydrothermal method. The steady-state kinetic constants and catalytic mechanism of Fe,N-CDs were investigated. The results showed that Fe,N-CDs exhibited high peroxidase-like activity and substrate affinity. Based on the ability of Fe,N-CDs to catalyze the decomposition of H_2_O_2_ and generate ·O^2-^, colorimetric detection of H_2_O_2_ was achieved by oxidizing TMB to ox-TMB with characteristic absorption at 652 nm. Indirect detection of Glu or Chol was accomplished by incorporating ChOx or GOx. Nanozyme-based ink was used to prepare testing paper as a paper-based sensor using inkjet printing. A simple visual detection method for Glu and Chol was developed using a smartphone. The synthesized nanozyme and developed visual detection platform exhibit great potential for the rapid and convenient detection of metabolite.

## Figures and Tables

**Table 1:** Detection of Glu or Chol in serum using the constructed colorimetric sensor.

Analyte	Sample	Added ^b^ (μM)	Found (μM)	Recovery (%)	RSD (%, n = 3)
		10.0	9.60	96.0	1.9
Glu	Serum ^a^	50.0	49.4	98.8	1.3
		100	98.4	98.4	0.3
	Serum ^a^	10.0	10.3	103	2.2
Chol		50.0	52.4	104	1.7
		100	97.5	97.5	0.9

^a^ Serum sample is diluted by a factor of 50. ^b^ The added concentration is obtained after dilution.

## Data Availability

The data presented in this study are available upon request from the corresponding author.

## Sample Availability

Samples of the compounds are available from the authors.
